# Rediscovery in science—a second eureka moment? A case in the neovascularization in cancer

**DOI:** 10.3332/ecancer.2018.ed79

**Published:** 2018-02-12

**Authors:** Denys N Wheatley

**Affiliations:** BioMedES UK, Leggat, Keithhall, Inverurie, Aberdeenshire, AB51 0LX, UK

**Keywords:** rediscovery, neovascularization, cancer, normalization

## Abstract

Rediscoveries are not uncommon. However, sometimes they can be more significant than confirmatory or extensions of existing findings, although many authors today refer to them as discoveries in their own right. This has led to papers repeatedly rehearsing the expression “we show here for the first time…”. When a finding has opened up a whole new field of research, this is more in line with a true discovery. When particular attention is drawn to such an event by editorials in widely read journals, such as *Nature*, its importance is bolstered. But if it turns out to be a rediscovery, the implications are considerable and the problem has to be brought to the attention not only of those in the same field of research, but to a wider audience to put the record straight. Consequently, acknowledgment of those who made the original discovery needs to be equally well publicised. A short discussion is presented of ways we might reduce the many claims of “new” discoveries that seem to be of considerable significance but are in fact rediscoveries.

## Introduction

For a variety of reasons, claims are often being made nowadays of discoveries that are not in fact new, or may only be corollaries to some more important finding that had been a real discovery many years (decades) ago. The phrase “we show for the first time…” resonates throughout the current scientific literature, which too often proves not to be the case.

## Discovery—its nature

Uncovering a phenomenon, finding a novel explanation, and introducing a new hypothesis largely constitutes the basis of all scientific endeavour. A discovery, whether small or large, is a unique event, a eureka moment; it is “when the penny drops”. Emotion suddenly wells up in the researcher, and all the hard work and effort that has led up to this moment is seen as being thoroughly worthwhile. This is the thrill, the reward, for work that has taken weeks, months, sometimes years and occasionally much of a lifetime (e.g. Higgs’ boson).

Science is all about understanding the universe from evidence gained through experimentation and by logical, rational and unemotional thinking. It is very largely a dispassionate activity. By consensus, new discoveries become part of the received wisdom and ought to fit comfortably (at least in due course) within the wider picture. They are not necessarily immutable, may have to be changed substantially, or often only apply under particular circumstances. They can be subsumed by some much broader hypothesis that we sometimes thereafter call theories, although still not necessarily tenable.

Some of the discoveries never seemed to fit within a broader context at the time (e.g. “jumping genes”) and only come back into mainstream when an apparently new idea arises because no good explanation was forthcoming at the time. In science more frequently than we realise, we are reading these days of discoveries that are in fact *re*discoveries. Since we all agree that there is little value in “reinventing the wheel”, why is this occurring more frequently these days?

## Rediscovery—a case in point concerning tumour neovascularization

Let is consider a recent example, the “rediscovery” of the nature of neovascularization of tumours [1–3], made worse in this case by an article in *Nature* drawing particular attention to it and dwelling on its significance [4]. Knowing that tumours have to co-opt a blood supply, it was noted that the vascular cells involved were often abnormal. This led to research directed at finding ways to “normalize” the situation. The research that followed went a good way to solving this problem, and to the development of dexrazoxane, which has anti-metastatic action and helps reduce cardiotoxicity often associated with a number of chemotherapeutic drugs used in cancer. The original discovery was made 40 years earlier than the new claims in refs [1, 2], and had been followed up subsequently in considerable detail [5, 6]. Asking researchers about situations of a similar nature, one begins to appreciate that in most cases (excepting for “world-shattering, massive ground-breaking” events), the authors simply have not read the literature thoroughly; indeed a quick search of the literature shows that there have been many papers on neovascularization in cancer and its normalization. Anything published a decade ago is nowadays considered “old hat”, a practice that is undoubtedly getting worse year by year.

## Technological advances—adding information to existing databases

The pace of technology seems to be making time march on ever faster, although it is generally accepted that discoveries are not generated in proportionate to the increasing investment and effort put into scientific research and technology. In the biomedical sciences, progress seems to have increased substantially because of modern day “kit culture”; every experiment can now be run faster and repeatedly by using pre-prepared reagents, kits and gadgets from manufacturers, making it much easier to spill out enormous amounts of data. Much time is spent simply finding information rather than testing hypotheses; and then more time has to be spent “data mining” and “number crunching”. This approach has as many disadvantages as advantages. First, the kit approach introduces a straightjacket; it narrows the limits of an experiment where other more suitable parameters might be as important and should be investigated. The kit might not even be the right one or the best method for a particular experiment, but it is often used because it happens to readily available (or the only one available). A kit can be very useful in a particular basic test, but it is not a case of one-size-fits-all; every experiment is unique and done under specific conditions, i.e. a kit might not be appropriate in one situation compared with another. Few researchers ever seem to adapt kits for their own specific circumstances, leading to that recurrent, unnecessary and jarring phrase “according to the manufacturer’s instructions” in the Materials and Methods sections of a high proportion of published papers, as if they might have ignored them or had used the kit inappropriately. The days of preparing one’s own requirements for truly critical experiments are almost a thing of the past.

Researchers also seem to have the wrong attitude in this regard; Karl Popper, the great science philosopher, espoused that one should not be experimenting to substantiate a hypothesis. He argued that you should strive to test your hypothesis to destruction; if it stands the onslaught, there *might* be some truth in it. Perhaps training in how to go about scientific research properly is inadequate. Experiments seem to be done largely to *prove* some point, often by selecting the findings that tend to support it, the rest being ignored or dismissed—they did not fit. The expression “this experiment didn’t work” or “something went wrong with it” can indicate this attitude.

## Conclusion

The above issues strongly suggest that the training of young scientists, particularly researchers and teachers, is not of the best, and only a few in a thousand usually have the inherent skills to make the kind of investigators who find truly new phenomena or solve a particularly thorny and perennial problem. Most researchers pick up how to go about their tasks by “osmosis”, copying their mentors and supervisors, some of whom might simply be passing on poor practice. The situation as far as I can assess after a lifetime of research in biomedicine has changed very little, if at all. It was quite poor in my early post-graduate years and certainly is no better today, a matter to which I have already drawn attention as far as scientific and medical communication is concerned [7].
